# Prognostic and predictive value of cathepsin X in serum from colorectal cancer patients

**DOI:** 10.1186/1471-2407-14-259

**Published:** 2014-04-13

**Authors:** Tjaša Vižin, Ib Jarle Christensen, Michael Wilhelmsen, Hans Jørgen Nielsen, Janko Kos

**Affiliations:** 1 Chair of Pharmaceutical Biology, Faculty of Pharmacy, University of Ljubljana, Ljubljana, Slovenia; 2 The Finsen Laboratory, Rigshospitalet, Copenhagen, Denmark; 3 Biotech Research and Innovation Centre (BRIC), University of Copenhagen, Copenhagen, Denmark; 4 Department of Surgical Gastroenterology, Hvidovre Hospital, Hvidovre, Denmark; 5 Institute of Clinical Medicine, Hvidovre Hospital, University of Copenhagen, Hvidovre, Denmark; 6 Department of Biotechnology, Jožef Stefan Institute, Ljubljana, Slovenia

**Keywords:** Cathepsin X, Colorectal cancer, Prognosis, Predictive marker, Serum biomarker

## Abstract

**Background:**

Cathepsin X is a cysteine protease involved in mechanisms of malignant progression. It is secreted from tumour cells as a proenzyme and may serve to predict the disease status and risk of death for cancer patients. In a previous, pilot, study on 77 colorectal patients we demonstrated the correlation of higher serum levels with shorter overall survival.

**Methods:**

264 patients with colorectal cancer were included in a prospectively accrued multi-centre observational cohort study with the aim of testing novel biomarkers. Blood samples were collected before preoperative large bowel endoscopy and total cathepsin X was measured in sera by ELISA. As a control group we selected at random 77 subjects who had no findings at endoscopy and reported no co-morbidity.

**Results:**

The mean level of cathepsin X in cancer patients did not differ from the control levels (23.4 ng/ml ± 6.4 SD vs. 18.8 ng/ml ± 11.4 SD, p > 0.05) and there was no association with age, gender, disease stage, tumour location or CEA. In univariate analysis no association between cathepsin X levels and overall survival was demonstrated for the entire set of patients, however, cathepsin X was associated with survival in a group of patients with local resectable disease (stages I-III) (HR = 1.69, 95% CI: 1.03-2.75, p = 0.03). For this group, multivariate Cox regression analysis showed an association (HR = 3.13, 95% CI: 1.37-7.18, p = 0.003) between high cathepsin X levels and shorter overall survival for patients who did not receive chemotherapy, whereas, for patients who received chemotherapy, there was no association between cathepsin X and survival (HR = 0.51, 95% CI: 0.20-1.33, p = 0.88).

**Conclusions:**

Association of cathepsin X levels with overall survival was not confirmed for an entire set of 264 colorectal patients, but for patients in stages I-III with local resectable disease. The significant association of cathepsin X with survival in a group of patients who received no chemotherapy and the absence of this association in the group who received chemotherapy, suggest the possible predictive value for response to chemotherapy. The results have to be confirmed in a further prospective study.

## Background

Discovery of molecules associated with colorectal cancer (CRC) whose levels may provide predictive and prognostic information, is important in order to improve treatment, optimize the quality of life and prolong survival of the patients. Several serum tumour markers are currently under evaluation, however, carcinoembryonic antigen (CEA) is the only one in regular clinical use for determining prognosis, for surveillance following curative resection and for monitoring therapy in advanced disease [1-4].

Cysteine cathepsins, a group of lysosomal cysteine proteases, have been reported as being involved in the development and progression of cancer. They are used as targets for developing new antitumour therapies. Their expression, protein and activity levels [5-7] have also been associated with prediction of prognosis in various cancer types, including CRC [8,9]. Cathepsins B and L, in particular, are the most extensively studied cysteine cathepsins in colorectal diseases. In colorectal carcinoma they are generally overexpressed and their ability to degrade the components of extracellular matrix has been related to tumour invasion, migration and metastasis [8,10]. Endogenous inhibitors of cysteine proteases, the cystatins, insufficiently impair the tumour associated activity of cathepsins; their lower levels in tumours, higher enzyme:inhibitor ratio and lower stability of the complex have been associated with cancer aggressiveness and patient survival [8,11].

In recent studies the potential of other cathepsins, exhibiting more specific functions and localisation than cathepsins B and L, have been evaluated. Cathepsin X (Cat X), for example, is expressed specifically in various cells of the immune system [12] and regulates important processes such as proliferation, maturation, migration and adhesion of immune cells, phagocytosis and signal transduction [13-15]. Elevated expression of Cat X has also been associated with the inflammatory processes in inflammatory related neurodegenerative disorders [16], *Helicobacter pylori* infection [17], multiple trauma [18] and tuberculosis [19]. However, there is increasing evidence that Cat X is also involved in malignant processes. The gene encoding Cat X is localized in chromosomal region 20q13, which is frequently amplified in several cancer types [20,21]. In lung tumours, immunohistochemical analysis revealed strong staining of Cat X in infiltrated immune cells, but very weak staining in tumour cells [12]. On the other hand, increased expression of Cat X was found in both tumour and immune cells of prostate [22] and gastric cancer [17] and in the most aggressive phenotypes of malignant melanoma [23]. Further, breast cancer transgenic mice with Cat X deficiency had a prolonged tumour-free period for breast cancer, unlike wild type mice [24]. Moreover, Cat X deficiency leads to accelerated cell senescence, which is a powerful tumour suppressing mechanism [25]. Cat X may also promote tumour processes by mechanisms typical of immune cells, such as binding and activation of integrin receptors [15] and modulation of CXCL-12 chemokine [26] and gamma enolase [16].

Reports evaluating the clinical relevance of Cat X are less numerous. Wang *et al*. demonstrated that increased Cat X mRNA levels in hepatocellular carcinomas correlate with advanced tumour stage [27]. In a pilot study our group showed significantly lower levels of active Cat X in sera of patients with inflammatory breast cancer than with non-inflammatory breast cancer [28]. However, no significant correlation with any clinico-pathological parameter could be demonstrated in epithelial ovarian cancer cyst fluid [29]. On the other hand, higher levels of Cat X (both pro-form and active mature form) in sera from 77 patients with CRC correlated significantly to shorter overall survival [30].

In the present study, the aim was to confirm the results of the pilot study with an extended cohort of CRC patients and, further, to evaluate the use of serum levels of total Cat X for selecting patients who could benefit from chemotherapy.

## Methods

### Patients

The study was approved by the Regional Ethical Committee of Copenhagen and The Danish Data Protection Agency. 264 subjects diagnosed with primary rectal or colon cancer and 77 controls were included in a multi-centre observational cohort study conducted at six Danish hospitals from October 2003 to December 2005. All gave informed consent to being included in the study. Cancer diagnoses were verified by histology of resected large bowel specimens. Patients who did not have the tumour removed for various reasons were classified according to the available clinical information and, as a result, exact tumour staging was not possible in 17 cases. As a control group, we selected subjects at random who had symptoms which could be due to CRC but exhibited no findings at endoscopy and reported no co-morbidity (healthy persons). Disease stage was based on the TNM-stage (International Union Against Cancer (UICC)). 26 patients with Stages I-III received adjuvant therapy whereas 33 patients with stage IV received palliative chemotherapy. It should be emphasized that patients were not randomized to adjuvant therapy and palliative chemotherapy but selected as part of the clinical decisions. Patient characteristics and applied first line therapies are given in Table 1.

**Table 1:** Patient characteristics and applied first line therapies

		**Subjects, n(%)**
Gender		
Female		105 (39.8)
Male		159 (60.2)
Age (years)		
70.3, 32.7 - 93.3 (median, range)		264 (100.0)
Localisation		
Left-sided colon cancer		97 (36.7)
Right-sided colon cancer		63 (23.9)
Rectal cancer		104 (39.4)
Stage		
I		42 (15.9)
II		73 (27.7)
III		63 (23.9)
IV		69 (26.1)
Not staged		17 (6.4)
Administered chemotherapy	Adjuvant Therapy (stages I-III)	Palliative Therapy (stage IV)

	Subjects, n (%)	Subjects, n (%)
5-FU	2 (1.1)	1 (1.4)
5-FU + Isovorin	7 (3.9)	4 (5.8)
Irinotecan + 5-FU	0 (0)	3 (4.3)
Oxaliplatin + 5-FU	7 (3.9)	2 (2.9)
Capecitabine	3 (1.7)	1 (1.4)
Oxaliplatin + Capecitabine	4 (2.2)	15 (21.7)
Other	1 (0.6)	4 (5.8)
Unknown	2 (1.1)	3 (4.3)
Total	26 (14.6)	33 (47.8)

### Sampling

Blood samples were collected from healthy persons and from CRC patients just before preoperative large bowel endoscopy according to a validated standard operating procedure. Blood was collected at moderate tourniquet pressure, in 10 ml serum tubes (Vacutainer® Becton-Dickinson, Mountain View, CA, USA), and spun for 10 minutes at 3000 *g* and 4°C within 1 hour of collection. Samples were immediately stored at −80°C.

### Cat X and CEA analysis

Human total Cat X was analysed by ELISA as described [12,30]. Goat polyclonal antibody (R&D SYSTEMS®, Minneapolis, USA) and horseradish peroxidase-conjugated 3B10 mouse monoclonal antibody, both recognizing pro- and mature Cat X, were used. Pro-Cat X, used as a standard, was prepared and characterized as described [31]. Serum samples were diluted in a 1:2 ratio with 2% BSA in 10 mM phosphate buffer (pH 7.2), 150 mM NaCl and 0.05% Tween® 20 (Sigma-Aldrich Chemie GmbH, Steinheim, Germany). The range of the calibration curve for Cat X extended from 1.0 to 32.5 ng/ml (see Additional file 1: Figure S1). To determine the linearity of the ELISA, serum samples were serially diluted in 2% BSA in 10 mM phosphate buffer to levels encompassing the range of the assay, and their linearity was evaluated by comparing the measured values with the calibration values. Dilutions of 4 serum samples showed a linear dose response parallel to the calibration curve in a dilution range 1:2 to 1:16 (see Additional file 2: Figure S2). To evaluate recovery rate, recombinant pro-Cat X at different concentrations was added to serum samples with known total Cat X concentrations. Recovery levels were determined by comparing the expected and observed concentrations. Recovery levels ranged from 87.0 to 100.6%. Mean recovery was 92.1%. The plot of expected vs. observed concentrations for Cat X ELISA is shown in (see Additional file 3: Figure S3). The intra-assay coefficient of variance (CV), determined by measuring 20 control replicates, was 6.5%. Inter-assay precision was derived by evaluating one high and one low control in duplicates in seven separate assays and was 7.9%. Detection limit of the assay, defined as the concentration corresponding to the mean absorbance of 10 replicates of zero standard plus 3 standard deviations (SDs) was determined to be 0.75 ng/ml.

CEA was determined using the Abbott ARCHITECT® i2000 automated immunoassay system utilising an on-market two-step dual monoclonal immunoassay (ARCHITECT® CEA assay). The determination was run at the Abbott Centre of Excellence research laboratory in Munich, Germany [32]. The assay precision is stated to be less than 8%, the mean recovery 99.9% and the detection limit better than 0.5 ng/ml (ARCHITECT® CEA assay datasheet).

All biomarker determinations were performed blinded to the technicians and to the study endpoint.

### Statistical analysis

Rank sum tests were used for test of location and Spearman’s rank correlation was used as a measure for association. Univariate and multivariate Cox regression models were used to assess the association of total Cat X and other clinicopathologic parameters with overall survival. Cat X was scored by its actual value on the log scale (base 2), which means that the hazard ratios are for a two fold difference in the marker level. The Cox model included an interaction term in order to test the hypothesis that the association of Cat X was not independent of chemotherapy received by the patient. Additionally, as in the pilot study, the median was used as a cut-off value in univariate analysis. CEA was dichotomized at 5 ng/ml. Model assessment of the proportionality assumption and linearity was done using Schoenfeld and martingale residuals as well as 10-fold internal cross validation [33]. The results of the model assessment confirmed the estimates. Graphical representations of survival probabilities were presented by Kaplan-Meier curves grouping continuous variables by their tertiles. P-values less than 5% were considered to be statistically significant. Statistical analysis was carried out using SAS (v9.2, SAS Institute, Cary, N.C., USA) and R (R Core Team (2013); R: A language and environment for statistical computing. R statistical Computing. Vienna. Austria. URL. http://www.R-project.org/).

The results of this project are reported in accordance with the REMARK guidelines [34].

## Results

### Levels of total Cat X and CEA and association with baseline variables

Total Cat X and CEA concentrations were measured in sera from 264 patients with CRC. Results and descriptive statistics are shown in Table 2. The mean level of total Cat X in 77 control samples was 18.8 ng/ml ± 11.4 SD. There were no significant differences in Cat X levels between patients and controls or between disease stages, cancer localisation and gender.

**Table 2:** Descriptive statistics for total Cat X serum concentrations

	**Median**	**Mean**	**SD**	**Minimum**	**Lower Quartile**	**Upper Quartile**	**Maximum**
	**(ng/ml)**	**(ng/ml)**	**(ng/ml)**	**(ng/ml)**	**(ng/ml)**	**(ng/ml)**	**(ng/ml)**
All samples	23.2	23.4	6.4	8.7	19.1	27.3	47.1
Stage^a^	p = 0.76						
I	23.4	24.7	7.4	12.6	20.3	28.5	44.5
II	23.4	22.9	5.6	10.9	18.6	26.6	35.4
III	22.4	22.8	6.4	8.7	19.1	27.5	38.1
IV	22.9	23.5	6.6	10.4	18.5	27.4	47.1
Cancer localisation	p = 0.36						
Left-sided colon cancer	23.3	24.2	6.0	12.3	20.1	27.8	40.0
Right-sided colon cancer	22.9	22.5	6.5	8.7	16.6	27.3	38.1
Rectal cancer	23.0	23.3	6.6	9.5	18.7	26.3	47.1
Gender	p = 0.49						
Female	23.3	23.9	6.2	11.8	19.2	27.4	44.5
Male	23.1	23.1	6.4	8.7	18.9	26.9	47.1

^a^17 patients were not staged.

Values of total serum Cat X for all samples, distributed by stage, cancer localisation and by gender.

Also, Spearman’s rank correlation between total Cat X concentrations, age and CEA showed no correlation (r = 0.08, p = 0.22; r = 0.02, p = 0.73). Median CEA was 3.7 ng/ml (range: 0.5-5046 ng/ml).

### Analysis of survival

In the univariate and multivariate Cox regression model, total Cat X was scored as a continuous variable on the log scale using base 2, which means that hazard ratios (HR) are for two fold differences. The median time from inclusion to end of study was 4.8 years (3.9-6.0).

Univariate analysis of Cat X, scored by its log transformed value in the entire set, showed no significant differences in overall survival (HR = 1.26; 95% CI: 0.82-1.92; p = 0.29), although high total Cat X levels were associated with poor survival. As the pilot study demonstrated a significant association between Cat X and overall survival, we additionally analysed the current study using the median as a threshold, as in the pilot study. Again, no significant difference was demonstrated (HR = 1.33; 95% CI: 0.95-1.86, p = 0.10). However, when patients in stages I-III were analysed in this way, the difference was significant (HR 1.69; 95% CI: 1.03-2.75; p = 0.03). Pre-specified multivariate analysis of time to death from all causes for patients in stages I-III, including the covariates gender, age (per 10 years), stage, tumour localisation, adjuvant chemotherapy, levels of CEA and total Cat X and interactions terms between chemotherapy and the biomarkers identified a subgroup of patients with a significant association with the outcome of the disease, as shown in Table 3.

**Table 3:** Multivariate analysis of total Cat X concentration and other potential factors for overall survival of 178 patients in stages I-III

	**HR**	**95% CI**	**p-value**
Gender			
Female	0.62	0.37-1.06	
			0.08
Male	1.0		
Age (per 10 years)	1.58	1.24-2.02	0.0003
Stage			
I	1.0		
II	1.23	0.58-2.62	0.01
III	2.74	1.27-5.92	
Localisation			
Right-sided colon cancer	1.00		
Left-sided colon cancer	1.18	0.61-2.28	0.79
Rectal Cancer	0.99	0.48-2.05	
CEA (≥ 5 ng/ml vs. < 5 ng/ml)			
No adjuvant chemotherapy	1.99	1.14-3.50	0.026
Adjuvant chemotherapy	0.42	0.13-1.39	0.60
Cat X (2-fold difference in HR)			
No adjuvant chemotherapy	3.13	1.37-7.18	0.003
Adjuvant chemotherapy	0.51	0.20-1.33	0.88

The estimated HR in multivariate analysis for total Cat X was 3.13 (95% CI: 1.37-7.18) for patients who did not receive chemotherapy and 0.51 (95% CI: 0.20-1.33) for those who received chemotherapy (interaction term p = 0.016). The latter means that patients who did not receive chemotherapy and had high total Cat X serum levels, had a significantly shorter survival (p = 0.003) comparable to that of the whole group of patients who had not undergone chemotherapy. A weaker, but still significant, association with survival within the same group of patients was also observed for CEA (p = 0.026). However, interaction between Cat X and CEA for this group of patients not receiving chemotherapy could not be demonstrated (p = 0.73), suggesting that the two markers are additive (i.e. not synergistic). The generalized concordance index was 0.75 (standard error (se) = 0.04), thus demonstrating reasonable discrimination in this model.

**Figure 1:**
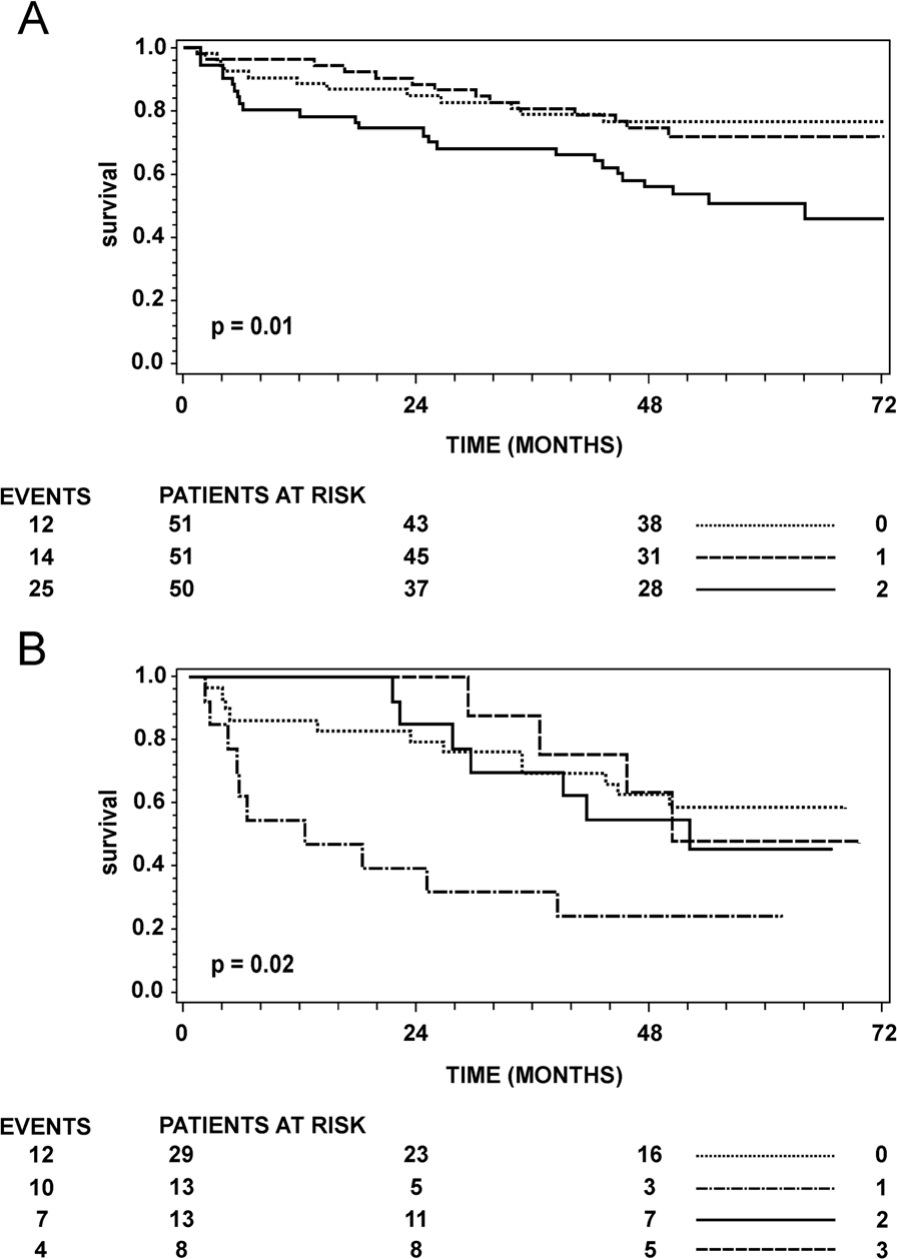
**Overall survival curves according to the total Cat X values in serum from patients with CRC.** The number of patients at risk at 0, 24 and 48 months is shown below the axis for each stratum and the number of deaths (events) is shown to the left. The difference between the strata was tested using the log rank test. **(A)** Kaplan-Meier estimates of survival for patients with stages I-III who did not receive chemotherapy. Cat X has been grouped using tertiles (denoted 0, 1, 2) **(B)** Kaplan-Meier estimates of survival for patients with stage III who, 0: did not receive chemotherapy and had low Cat X, 1: did not receive chemotherapy and had high Cat X, 2: received chemotherapy and had low Cat X, 3: received chemotherapy and had high Cat X.

Within the group of patients who received therapy there was no significant difference in overall survival between patients with low and high total Cat X. These results suggest the potential of total Cat X to predict the response to chemotherapy. Kaplan-Meier curves provide a similar result for patients with stages I-III, who did not receive chemotherapy (Figure 1A). Patients with total Cat X levels in the third tertile exhibited a significantly shorter overall survival than those from the first and second tertiles (p = 0.01). When Kaplan-Meier estimates of survival are stratified to stage III, again patients who did not receive chemotherapy with Cat X in the third tertile showed significantly shorter overall survival than those with low Cat X, whereas stage III patients who received chemotherapy exhibited no significant difference with regard to Cat X levels (Figure 1B). Inclusion of specific first line chemotherapies in multivariate analyses did not demonstrate significant interactions between specific therapy and Cat X (data not shown). Therapies involving 5-Fluorouracil (5-FU), Oxaliplatin and Capecitabine were tested in stages I-III and stage IV. However this study is not designed or powered to address this question.

No significant effect of Cat X on survival could be demonstrated for stage IV, nor for univariate (HR = 1.02; 95% CI: 0.54-1.93; p = 0.95) or multivariate (HR = 0.68; 95% CI: 0.34-1.39; p = 0.29) analysis. Including patients not staged, a similar result was found (data not shown). 33 (48%) patients with stage IV received palliative chemotherapy but no significant interaction between total Cat X and chemotherapy could be demonstrated (p = 0.21).

## Discussion

Colorectal cancer is the second leading cause of cancer-related death in Europe and the US. About half of the patients develop recurrent disease within 3 to 5 years of resection of the primary tumour, and they are candidates for systemic chemotherapy. The availability of validated biological markers for selection of chemotherapy and prediction of treatment efficacy, together with monitoring, may increase patient survival. In this study we identified serum Cat X as a potential tumour marker in CRC, based on the demonstrated correlation of its high levels with shorter patient overall survival and the association with the adjuvant chemotherapy.

In our previous (pilot) study on 77 CRC patients [30] we found that levels of total Cat X in sera from patients with CRC were not significantly higher than those in groups of healthy persons, patients with adenomas or patients with non-neoplastic findings, all matched with CRC patients for age and gender. The mean level of total Cat X, determined in sera from the 264 CRC patients included in this confirmation study, also does not differ statistically from that in healthy persons (mean 23.4 ng/mL ± 6.4 SD vs. 18.8 ng/ml ± 11.4 SD, p > 0.05). As in the pilot study, there is no significant association with patient age or gender, tumour stage or location and CEA. In a univariate Cox regression model, the levels of total Cat X evaluated in this study did not confirm the significant relation to overall survival found in the pilot study [30], although the higher levels were associated with poor survival. When, as in the pilot study, the median was used as a cut off value, a similar result was obtained for the entire set of patients whereas, for patients with local resectable disease (stages I-III), the relation to overall survival was significant. In contrast, for patients with metastatic disease (stage IV), there was no relation between Cat X and overall survival. In multivariate analysis, including patients with stages I-III, a strong association (HR = 3.13) between high total Cat X levels and shorter overall survival was observed for the group of patients who did not receive adjuvant chemotherapy whereas, within the group of patients who received chemotherapy, there was no association between total Cat X and survival (HR = 0.51). Patient age, stage and CEA also contributed to the prognosis in multivariate analysis, whereas gender and location were not significant. The significant interaction between Cat X and adjuvant chemotherapy suggests its possible predictive value, since the data suggest that patients with high levels of Cat X benefit from the therapy.

Several mechanisms have been suggested that could link higher activity and/or concentration of Cat X with the progression of malignant disease. Cat X can be present in sera from healthy persons due to constitutive secretion by leukocytes during normal physiological immune cell activation and turnover [18]. Increased secretion of pro-Cat X could reflect the overexpression of Cat X in tumour and tumour associated immune cells, as observed in several cancer types. Secreted pro-Cat X most probably remains in the extracellular space, however, it can be activated by membrane bound cathepsin L [35] or surface heparan sulphate proteoglycans [13]. The activation by cathepsin L can also take place in endosomal/lysosomal vesicles during their translocation toward the plasma membrane [15,35]. Pro-Cat X can modulate cell adhesion and motility through interaction of its pro-region with heparan sulphate proteoglycans [13] and integrin receptors [36-38], while active Cat X can regulate cell functions through cleavage of C-terminal amino acids of chemokine CXCL-12 [26], beta-2 chain of integrin receptors [36], gamma-enolase (neuronal specific enolase - NSE) [16] and profilin [39,40]. Therefore, both forms could promote tumour progression processes, resulting in enhanced adhesion and migration of tumour cells, induced epithelial-mesenchymal transition [27] and decreased cell senescence [25]. Results obtained on transgenic animals further confirm the active role of Cat X in tumour progression. It was suggested that Cat X, not being dependent on the degradation of extracellular matrix, a typical event for cathepsin B, may compensate the malignant effects of cathepsin B [24,41] by changing the migration mode of tumour cells. Only mice with excluded expression of both cathepsin B and Cat X exhibited significantly lower tumour growth and metastasis formation. The relation between Cat X levels and response to therapy has not been reported, however, anticancer drugs may interfere with Cat X targets and processes, also regulating its expression and secretion.

From previous studies it is not clear whether Cat X is involved in early or late stages of cancer. The results by Sevenich *et al*. [24], using a PymT-induced metastatic breast cancer mouse model, showed that Cat X was related to the initial stages of the malignant process and less with tumour progression and metastasis. On the other hand, Hidaka *et al.* showed that Cat X could be involved in the later stages of cancer, since amplification of the region encoding for Cat X correlated with the metastatic potential and tumour progression in CRC [20]. A similar possibility was explored by Wang *et al.*[27], who showed that increased Cat X expression strongly correlated with advanced clinical stage, and induced metastatic potential and shorter overall survival of patients with hepatocellular carcinoma. Moreover, it was recently demonstrated that, in adenocarcinoma of the pancreatic duct, S100P-binding protein inversely regulates Cat X expression – lower levels of S100P-binding protein increased Cat X, cell adhesion and metastatic potential of pancreatic cancer cells [42]. Our findings are in accord with those of a recent study on lung cancer patients by Zhang *et al*., which also shows that higher Cat X serum levels are associated with shorter patient survival. However, lung cancer patients had significantly higher Cat X levels than healthy controls, which was not the case in our study [43]. Our combined results support the studies revealing the association of Cat X expression with the progression of CRC, since they provide the correlation of high levels of Cat X with shorter overall survival of CRC patients.

## Conclusions

Our study confirms the previous results relating higher Cat X levels to progression of cancer and shorter overall survival of CRC patients. Although total Cat X serum levels did not differ between the entire group of patients and controls, a significant difference was found in the group of patients who did not receive adjuvant chemotherapy: patients with high total Cat X serum levels had significantly shorter overall survival than those with low levels. For patients who received adjuvant chemotherapy no difference in overall survival was observed between those groups with low and high total Cat X. Thus, total Cat X could be useful as a predictive, blood-based tumour marker that may allow selection of patients who could benefit from adjuvant chemotherapy. However, the results must be confirmed in a prospective clinical study.

## Abbreviations

Cat X: Cathepsin X; CEA: Carcinoembryonic antigen; CI: Confidence interval; CRC: Colorectal cancer; CV: Coefficient of variance; CXCL-12: C-X-C motif chemokine 12; 5-FU: 5-Fluorouracil; HR: Hazard ratio; PymT: Polyomavirus middle T oncogene; SD: Standard deviation.

## Competing interest

The authors declare that they have no competing interests.

## Authors’ contributions

TV designed the study, carried out the immunochemical analyses and drafted the manuscript. IJC analysed data, performed the statistical analysis and helped draft the manuscript. MW collected clinical data and participated in the evaluation of adjuvant chemotherapy. HJN participated in the design of the study and coordination of sampling and clinical evaluation. JK conceived the study, participated in coordination and helped draft the manuscript. All authors read and approved the final manuscript.

## Pre-publication history

The pre-publication history for this paper can be accessed here:

http://www.biomedcentral.com/1471-2407/14/259/prepub

## Supplementary Material

Additional file 1: Figure S1 Calibration curve for human total Cat X. Click here for file

Additional file 2: Figure S2 Linearity of the total Cat X ELISA. Dilution curves of four sera from CRC patients. Sera were serially diluted (1/2, 1/4, 1/8, 1/16). Click here for file

Additional file 3: Figure S3 Analytical recovery. Observed total Cat X concentrations vs. expected total Cat X concentrations in serum samples from patients with CRC. Three different amounts of pro-Cat X were added to known amounts of total Cat X in two serum samples. Click here for file

## References

[B1] Sturgeon CM Duffy MJ Stenman UH Lilja H Brunner N Chan DW Babaian R Bast Jr. RC Dowell B Esteva FJ Haglund C Harbeck N Hayes DF Holten-Andersen M Klee GG Lamerz R Looijenga LH Molina R Nielsen HJ Rittenhouse H Semjonow A Shih Ie M Sibley P Soletormos G Stephan C Sokoll L Hoffman BR Diamandis EP National Academy of Clinical Biochemistry laboratory medicine practice guidelines for use of tumor markers in testicular, prostate, colorectal, breast, and ovarian cancers Clin Chem 2008 54 e11 e79 10.1373/clinchem.2008.105601 19042984

[B2] Booth RA Minimally invasive biomarkers for detection and staging of colorectal cancer Cancer Lett 2007 249 87 96 10.1016/j.canlet.2006.12.021 17275174

[B3] Nielsen HJ Brunner N Frederiksen C Lomholt AF King D Jorgensen LN Olsen J Rahr HB Thygesen K Hoyer U Laurberg S Christensen IJ Danish-Australian Endoscopy Study Group On Colorectal Cancer D, Danish Colorectal Cancer Cooperative G Plasma tissue inhibitor of metalloproteinases-1 (TIMP-1): a novel biological marker in the detection of primary colorectal cancer. Protocol outlines of the Danish-Australian endoscopy study group on colorectal cancer detection Scand J Gastroenterol 2008 43 242 248 10.1080/00365520701523439 18224568

[B4] Nielsen HJ Christensen IJ Brunner N A novel prognostic index in colorectal cancer defined by serum carcinoembryonic antigen and plasma tissue inhibitor of metalloproteinases-1 Scand J Gastroenterol 2010 45 200 207 10.3109/00365520903429406 20095885

[B5] Turk V Kos J Turk B Cysteine cathepsins (proteases)–on the main stage of cancer? Cancer Cell 2004 5 409 410 10.1016/S1535-6108(04)00117-5 15144947

[B6] Strojan P Cysteine cathepsins and stefins in head and neck cancer: an update of clinical studies Radiol Oncol 2008 42 69 81

[B7] Ardebili SY Zajc I Gole B Campos B Herold-Mende C Drmota S Lah TT CD133/prominin1 is prognostic for GBM patient’s survival, but inversely correlated with cysteine cathepsins’ expression in glioblastoma derived spheroids Radiol Oncol 2011 45 102 115 22933943 10.2478/v10019-011-0015-6 PMC3423731

[B8] Kos J Lah TT Cysteine proteinases and their endogenous inhibitors: target proteins for prognosis, diagnosis and therapy in cancer (review) Oncology Rep 1998 5 1349 1361 10.3892/or.5.6.1349 9769367

[B9] Troy AM Sheahan K Mulcahy HE Duffy MJ Hyland JM O’Donoghue DP Expression of Cathepsin B and L antigen and activity is associated with early colorectal cancer progression Eur J Cancer 2004 40 1610 1616 10.1016/j.ejca.2004.03.011 15196548

[B10] Kuester D Lippert H Roessner A Krueger S The cathepsin family and their role in colorectal cancer Pathol Res Pract 2008 204 491 500 10.1016/j.prp.2008.04.010 18573619

[B11] Zore I Krasovec M Cimerman N Kuhelj R Werle B Nielsen HJ Brunner N Kos J Cathepsin B/cystatin C complex levels in sera from patients with lung and colorectal cancer Biol Chem 2001 382 805 810 11517934 10.1515/BC.2001.097

[B12] Kos J Sekirnik A Premzl A Zavasnik Bergant V Langerholc T Turk B Werle B Golouh R Repnik U Jeras M Turk V Carboxypeptidases cathepsins X and B display distinct protein profile in human cells and tissues Exp Cell Res 2005 306 103 113 10.1016/j.yexcr.2004.12.006 15878337

[B13] Nascimento FD Rizzi CCA Nantes IL Stefe L Turk B Carmona AK Nader HB Juliano L Tersariol ILS Cathepsin X binds to cell surface heparan sulfate proteoglycans Arch Biochem Biophys 2005 436 323 332 10.1016/j.abb.2005.01.013 15797245

[B14] Obermajer N Jevnikar Z Doljak B Sadaghiani AM Bogyo M Kos J Cathepsin X-mediated beta2 integrin activation results in nanotube outgrowth Cell Mol Life Sci 2009 66 1126 1134 10.1007/s00018-009-8829-8 19194656 PMC11131452

[B15] Kos J Jevnikar Z Obermajer N The role of cathepsin X in cell signaling Cell Adh Migr 2009 3 164 166 10.4161/cam.3.2.7403 19262176 PMC2679876

[B16] Obermajer N Doljak B Jamnik P Fonovic UP Kos J Cathepsin X cleaves the C-terminal dipeptide of alpha- and gamma-enolase and impairs survival and neuritogenesis of neuronal cells Int J Biochem Cell Biol 2009 41 1685 1696 10.1016/j.biocel.2009.02.019 19433310

[B17] Krueger S Kalinski T Hundertmark T Wex T Kuster D Peitz U Ebert M Nagler DK Kellner U Malfertheiner P Naumann M Rocken C Roessner A Up-regulation of cathepsin X in Helicobacter pylori gastritis and gastric cancer J Pathol 2005 207 32 42 10.1002/path.1820 16025436

[B18] Nagler DK Lechner AM Oettl A Kozaczynska K Scheuber HP Gippner-Steppert C Bogner V Biberthaler P Jochum M An enzyme-linked immunosorbent assay for human cathepsin X, a potential new inflammatory marker J Immunol Methods 2006 308 241 250 10.1016/j.jim.2005.11.002 16376371

[B19] Baker AR Zalwango S Malone LL Igo RP Jr Qiu F Nsereko M Adams MD Supelak P Mayanja-Kizza H Boom WH Stein CM Genetic susceptibility to tuberculosis associated with cathepsin Z haplotype in a Ugandan household contact study Hum Immunol 2011 72 426 430 10.1016/j.humimm.2011.02.016 21354459 PMC3078986

[B20] Hidaka S Yasutake T Takeshita H Kondo M Tsuji T Nanashima A Sawai T Yamaguchi H Nakagoe T Ayabe H Tagawa Y Differences in 20q13.2 copy number between colorectal cancers with and without liver metastasis Clin Cancer Res 2000 6 2712 2717 10914715

[B21] Bar-Shira A Pinthus JH Rozovsky U Goldstein M Sellers WR Yaron Y Eshhar Z Orr-Urtreger A Multiple genes in human 20q13 chromosomal region are involved in an advanced prostate cancer xenograft Cancer Res 2002 62 6803 6807 12460888

[B22] Nagler DK Kruger S Kellner A Ziomek E Menard R Buhtz P Krams M Roessner A Kellner U Up-regulation of cathepsin X in prostate cancer and prostatic intraepithelial neoplasia Prostate 2004 60 109 119 10.1002/pros.20046 15162377

[B23] Rumpler G Becker B Hafner C McClelland M Stolz W Landthaler M Schmitt R Bosserhoff A Vogt T Identification of differentially expressed genes in models of melanoma progression by cDNA array analysis: SPARC, MIF and a novel cathepsin protease characterize aggressive phenotypes Exp Dermatol 2003 12 761 771 10.1111/j.0906-6705.2003.00082.x 14714555

[B24] Sevenich L Schurigt U Sachse K Gajda M Werner F Muller S Vasiljeva O Schwinde A Klemm N Deussing J Peters C Reinheckel T Synergistic antitumor effects of combined cathepsin B and cathepsin Z deficiencies on breast cancer progression and metastasis in mice Proc Natl Acad Sci U S A 2010 107 2497 2502 10.1073/pnas.0907240107 20133781 PMC2823914

[B25] Kraus S Bunsen T Schuster S Cichon MA Tacke M Reinheckel T Sommerhoff CP Jochum M Nagler DK Cellular senescence induced by cathepsin X downregulation Eur J Cell Biol 2011 90 678 686 10.1016/j.ejcb.2011.03.008 21616554

[B26] Staudt ND Aicher WK Kalbacher H Stevanovic S Carmona AK Bogyo M Klein G Cathepsin X is secreted by human osteoblasts, digests CXCL-12 and impairs adhesion of hematopoietic stem and progenitor cells to osteoblasts Haematologica 2010 95 1452 1460 10.3324/haematol.2009.018671 20494937 PMC2930944

[B27] Wang J Chen LL Li Y Guan XY Overexpression of cathepsin Z contributes to tumor metastasis by inducing epithelial-mesenchymal transition in hepatocellular carcinoma PLoS One 2011 6 e24967 10.1371/journal.pone.0024967 21966391 PMC3178578

[B28] Decock J Obermajer N Vozelj S Hendrickx W Paridaens R Kos J Cathepsin B, cathepsin H, cathepsin X and cystatin C in sera of patients with early-stage and inflammatory breast cancer Int J Biol Markers 2008 23 161 168 18949742 10.1177/172460080802300305

[B29] Kolwijck E Kos J Obermajer N Span PN Thomas CM Massuger LF Sweep FC The balance between extracellular cathepsins and cystatin C is of importance for ovarian cancer Eur J Clin Invest 2010 40 591 599 10.1111/j.1365-2362.2010.02305.x 20482593

[B30] Vizin T Christensen IJ Nielsen HJ Kos J Cathepsin X in serum from patients with colorectal cancer: relation to prognosis Radiol Oncol 2012 46 207 212 23077459 10.2478/v10019-012-0040-0 PMC3472949

[B31] Fonovic UP Kos J Efficient removal of cathepsin L from active cathepsin X using immunoprecipitation technique Acta Chim Slov 2009 56 985 988

[B32] Nielsen HJ Brunner N Jorgensen LN Olsen J Rahr HB Thygesen K Hoyer U Laurberg S Stieber P Blankenstein MA Davis G Dowell BL Christensen IJ Plasma TIMP-1 and CEA in detection of primary colorectal cancer: a prospective, population based study of 4509 high-risk individuals Scand J Gastroenterol 2011 46 60 69 10.3109/00365521.2010.513060 20799911

[B33] Harrell FE Jr Lee KL Mark DB Multivariable prognostic models: issues in developing models, evaluating assumptions and adequacy, and measuring and reducing errors Stat Med 1996 15 361 387 10.1002/(SICI)1097-0258(19960229)15:4<361::AID-SIM168>3.0.CO;2-4 8668867

[B34] Altman DG McShane LM Sauerbrei W Taube SE Reporting recommendations for tumor marker prognostic studies (REMARK): explanation and elaboration PLoS Med 2012 9 e1001216 10.1371/journal.pmed.1001216 22675273 PMC3362085

[B35] Nagler DK Zhang RL Tam W Sulea T Purisima EO Menard R Human cathepsin X: A cysteine protease with unique carboxypeptidase activity Biochemistry 1999 38 12648 12654 10.1021/bi991371z 10504234

[B36] Obermajer N Premzl A Zavasnik Bergant T Turk B Kos J Carboxypeptidase cathepsin X mediates beta2-integrin-dependent adhesion of differentiated U-937 cells Exp Cell Res 2006 312 2515 2527 10.1016/j.yexcr.2006.04.019 16774752

[B37] Obermajer N Repnik U Jevnikar Z Turk B Kreft M Kos J Cysteine protease cathepsin X modulates immune response via activation of beta2 integrins Immunology 2008 124 76 88 10.1111/j.1365-2567.2007.02740.x 18194276 PMC2434384

[B38] Lechner AM Assfalg-Machleidt I Zahler S Stoeckelhuber M Machleidt W Jochum M Nagler DK RGD-dependent binding of procathepsin X to integrin alphavbeta3 mediates cell-adhesive properties J Biol Chem 2006 281 39588 39597 10.1074/jbc.M513439200 17065156

[B39] Pecar Fonovic U Jevnikar Z Rojnik M Doljak B Fonovic M Jamnik P Kos J Profilin 1 as a target for cathepsin X activity in tumor cells PLoS One 2013 8 e53918 10.1371/journal.pone.0053918 23326535 PMC3542269

[B40] Skvortsova I Profilin 1: do we have a novel proteome-found biomarker predicting response to anticancer therapy? Proteomics 2013 13 2069 2071 10.1002/pmic.201300165 23677805

[B41] Vasiljeva O Papazoglou A Kruger A Brodoefel H Korovin M Deussing J Augustin N Nielsen BS Almholt K Bogyo M Peters C Reinheckel T Tumor cell-derived and macrophage-derived cathepsin B promotes progression and lung metastasis of mammary cancer Cancer Res 2006 66 5242 5250 10.1158/0008-5472.CAN-05-4463 16707449

[B42] Lines KE Chelala C Dmitrovic B Wijesuriya N Kocher HM Marshall JF Crnogorac-Jurcevic T S100P-binding protein, S100PBP, mediates adhesion through regulation of cathepsin Z in pancreatic cancer cells Am J Pathol 2012 180 1485 1494 10.1016/j.ajpath.2011.12.031 22330678

[B43] Zhang X Hou Y Niu Z Li W Meng X Zhang N Yang S Clinical significance of detection of cathepsin X and cystatin C in the sera of patients with lung cancer Zhongguo Fei Ai Za Zhi 2013 16 411 416 23945244 10.3779/j.issn.1009-3419.2013.08.04 PMC6000661

