# Supplementary figures and images for: Prognostic and predictive value of cathepsin X in serum from colorectal cancer patients
Source: BMC Cancer. 2014 Apr 13;14:259. doi: 10.1186/1471-2407-14-259 (PMC4021260; doi:10.1186/1471-2407-14-259)

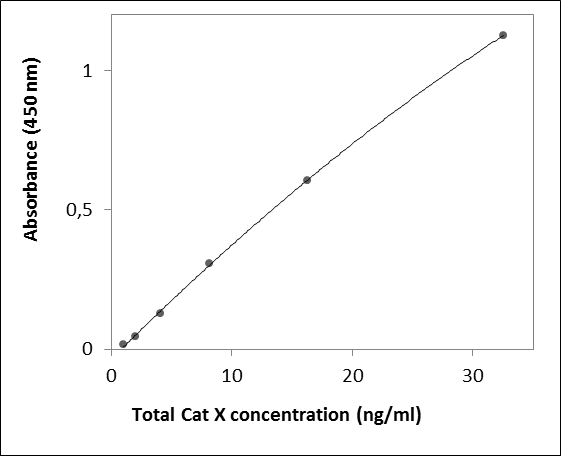

Supplement: Additional file 1: Figure S1 — Calibration curve for human total Cat X. [file 1471-2407-14-259-S1.tiff]

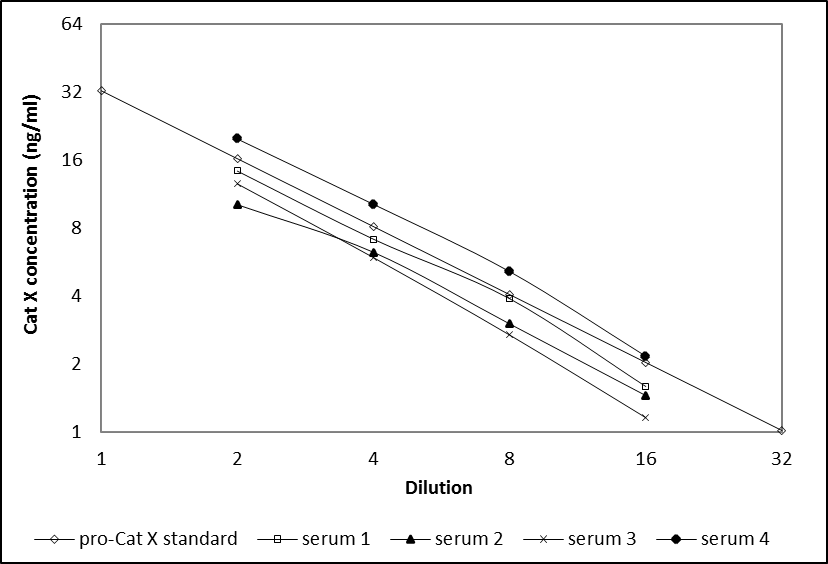

Supplement: Additional file 2: Figure S2 — Linearity of the total Cat X ELISA. Dilution curves of four sera from CRC patients. Sera were serially diluted (1/2, 1/4, 1/8, 1/16). [file 1471-2407-14-259-S2.tiff]

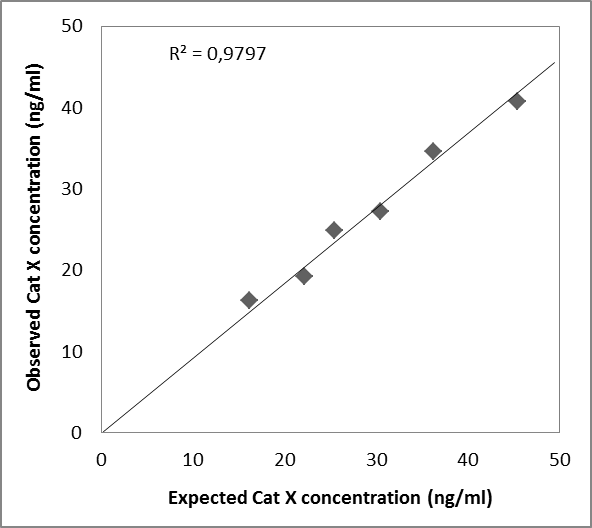

Supplement: Additional file 3: Figure S3 — Analytical recovery. Observed total Cat X concentrations vs. expected total Cat X concentrations in serum samples from patients with CRC. Three different amounts of pro-Cat X were added to known amounts of total Cat X in two serum samples. [file 1471-2407-14-259-S3.tiff]
